# Human Antibody Responses to Avian Influenza A(H7N9) Virus, 2013

**DOI:** 10.3201/eid2002.131094

**Published:** 2014-02

**Authors:** Li Guo, Xi Zhang, Lili Ren, Xuelian Yu, Lijuan Chen, Hongli Zhou, Xin Gao, Zheng Teng, Jianguo Li, Jiayu Hu, Chao Wu, Xia Xiao, Yiyi Zhu, Quanyi Wang, Xinghuo Pang, Qi Jin, Fan Wu, Jianwei Wang

**Affiliations:** MOH Key Laboratory of Systems Biology of Pathogens, Beijing, People’s Republic of China (L. Guo, L. Ren, J. Li, Q. Jin, J. Wang);; Institute of Pathogen Biology, Beijing (L. Guo, L. Ren, H. Zhou, X. Gao, J. Li, C. Wu, X. Xiao, Q. Jin, J. Wang);; Shanghai Municipal Center for Disease Control and Prevention, Shanghai, People’s Republic of China (X. Zhang, X. Yu, Z. Teng, J. Hu, Y. Zhu, F. Wu);; Beijing Municipal Center for Disease Control and Prevention, Beijing (L. Chen, Q. Wang, X. Pang)

**Keywords:** avian influenza virus, H7N9, antibody responses, neutralizing antibody, hemagglutination inhibition assay, avidity, viruses, influenza, human

## Abstract

Understanding host antibody response is crucial for predicting disease severity and for vaccine development. We investigated antibody responses against influenza A(H7N9) virus in 48 serum samples from 21 patients, including paired samples from 15 patients. IgG against subtype H7 and neutralizing antibodies (NAbs) were not detected in acute-phase samples, but ELISA geometric mean titers increased in convalescent-phase samples; NAb titers were 20–80 (geometric mean titer 40). Avidity to IgG against subtype H7 was significantly lower than that against H1 and H3. IgG against H3 was boosted after infection with influenza A(H7N9) virus, and its level in acute-phase samples correlated with that against H7 in convalescent-phase samples. A correlation was also found between hemagglutinin inhibition and NAb titers and between hemagglutinin inhibition and IgG titers against H7. Because of the relatively weak protective antibody response to influenza A(H7N9), multiple vaccinations might be needed to achieve protective immunity.

In March 2013, an emerging virus, influenza A(H7N9), of novel avian origin was identified in humans in China (*1**,**2*). As of August 1, 2013, a total of 134 infections and 45 deaths had been reported (www.moh.gov.cn/zhuzhan/yqxx/201309/1f465a32fa8b476c93a4075e07742685.shtml). Other avian influenza virus subtypes, including H5N1, H6N1, H7N1, H7N2, H7N3, H7N7, H9N2, and H10N7 (*3**–**10*), have been transmitted directly from poultry to humans and, with the exception of 1 subtype H7N7 infection that caused death, cause mild symptoms (*5*–*8*). In contrast, most subtype H7N9 infections cause severe lower respiratory infections; estimated mortality rate is 30% (*2*). Clinical observations indicate that subtype H7N9 tends to cause severe symptoms in elderly (>60 years of age) patients, and these symptoms last longer (median 41.7 days) than those caused by subtype H5N1 infections (median 18.7 days) (*11*). The potential for subtype H7N9 virus to cause a pandemic among humans raises great public health concern (*12**–**14*).

Host immunity affects disease severity, disease duration, and vaccine response with regard to influenza virus infections and viral pathogenesis. Neutralizing antibodies (NAbs) are one of the most critical factors in virus clearance and disease outcome (*15**,**16*). NAbs, whether given as postexposure treatment or preexposure prophylaxis, protect animals from infection (*17*). In contrast, low-avidity antibodies against influenza virus might have adverse effects during infection (*15*). To determine human antibody responses to influenza A(H7N9) virus, we examined serum samples from infected patients.

## Methods

### Patients and Samples

From March 30 through August 12, 2013, a total of 48 serum samples were collected from 21 patients with influenza A(H7N9) virus infection in Shanghai and Beijing, China: 21 acute-phase (collected <7 days after symptom onset), 18 convalescent-phase (collected >14 days after symptom onset), and another 9 samples (collected 102–125 days after symptom onset from patients from whom paired samples were collected). Because of some patient deaths, ethical considerations, and the difficulty of following up with patients in isolation units, most convalescent-phase samples were collected from patients immediately before hospital discharge. Hence, paired convalescent-phase serum samples were obtained from 15 patients at various times (17–37 days after symptom onset). Only acute-phase serum samples were obtained from the other 6 patients. For the 15 patients for whom paired serum samples were available, 2 convalescent-phase samples were collected from 3 patients at different times (Table 1) and 1 convalescent-phase sample was collected from each of the other 12 patients. To trace serum conversion, an additional 9 serum samples were collected 102–125 days after symptom onset from the patients for whom paired serum samples were available.

**Table 1:** Clinical characteristics of patients infected with influenza A(H7N9) virus, China, 2013*

Patient no.	Age, y/sex	Concurrent conditions	Days of admission after symptom onset	ICU	Complications	Mechanical ventilation	Days to commencing antiviral treatment after admission	Oseltamivir	Outcome
1	66/M	Arthritis, prostatitis	6	No	No	No	6	Yes	R
2	73/M	Chronic bronchitis	7	No	No	No	8	Yes	R
3	67/M	Hypertension	5	No	No	No	5	Yes	R
4	62/M	Hypertension	4	NA	No	No	7	Yes	R
5	76/F	Diabetes mellitus, hypertension, coronary disease	7	NA	No	Yes	No	No	R
6	81/F	Coronary disease, rheumatoid disease	4	NA	No	No	6	Yes	R
7	83/F	Diabetes mellitus, hypertension	6	Yes	Respiratory failure, MODS	Yes	8	Yes	D
8	68/M	Diabetes mellitus, hypertension	6	No	No	No	7	Yes	R
9	53/M	Diabetes mellitus	7	Yes	Respiratory failure	Yes	8	Yes	R
10	54/M	Hypertension	5	No	No	No	5	Yes	R
11	79/M	Hypertension	5	NA	No	Yes	9	Yes	R
12	47/M	None	6	No	No	Yes	6	Yes	R
13	75/F	Diabetes mellitus, hypertension	6	NA	No	Yes	No	No	R
14	61/F	None	3	Yes	Respiratory failure, MODS	Yes	NA	Yes	D
15	7/F	None	1	Yes	No	Yes	1	Yes	R
16†	4/M	None	6	No	No	No	6	Yes	R
17	52/M	Thyroid tumor	7	No	Respiratory failure	Yes	7	Yes	D
18	77/M	Hypertension, atrial fibrillation	5	NA	NA	Yes	5	Yes	D
19	56/M	NA	3	Yes	ARDS, DIC, hemorrhagic shock	Yes	3	Yes	D
20	89/M	Hypertension, diabetes mellitus	4	NA	MODS	No	No	No	D
21	80/M	Coronary disease, cirrhosis	7	NA	Respiratory failure, acute heart failure	Yes	7	Yes	D

*ICU, admission to intensive care unit; R, recovered; NA, not available; D, died; MODS, multiple organ dysfunction syndrome: ARDS, acute respiratory distress syndrome; DIC, disseminated (or diffuse) intravascular coagulation.

For confirmation of influenza A(H7N9) virus infection, respiratory samples (throat swab or sputum samples) were obtained from each patient at the time of hospital admission. From these samples, total RNA was extracted by using a QIAamp Viral RNA Mini Kit (QIAGEN, Hilden, Germany); RNA for subtype H7N9 was detected by a real-time reverse transcription PCR (RT-PCR) kit provided by the Chinese National Influenza Center, Chinese Center for Disease Control and Prevention. In accordance with protocol recommendations, specimens were considered positive if the cycle threshold was <38.0. Clinical data were retrieved through a retrospective review of the medical records for each patient. The severity of subtype H7N9 infections was determined according to the guidelines for the management of pneumonia in children and adults (*18**,**19*).

For use as controls, 100 serum samples were collected from healthy blood donors in Beijing, China, from October through December 2008, and another 77 serum samples were collected from open-market poultry workers in Shanghai, China, in May and June 2013. All procedures involving subtype H7N9 virus were performed in a Biosafety Level 3 laboratory. 

Written, informed consent was obtained from all participants. The study was approved by the required ethics review boards.

### ELISA

Bound IgG titers for serum samples were evaluated by using an influenza virus hemagglutinin IgG ELISA. The hemagglutinin proteins of subtype H7N9 (A/Anhui/1/2013[H7N9]) and of seasonal subtypes H1N1 (A/California/04/2009[H1N1]) and H3N2 (A/Brisbane/10/2007[H3N2]) viruses were expressed in human embryonic kidney 293 cells (H3 hemagglutinin) or a baculovirus-insect cell system (H1 and H7 hemagglutinin) and purified by using 6× His tag (Sino Biologic, Beijing, China). The hemagglutinin activities of the recombinant H1, H3, and H7 hemagglutinin proteins were confirmed by using an Octet system (FortéBIO, Menlo Park, CA, USA) (data not shown). Recombinant H1, H3, and H7 hemagglutinins were used as coating antigens in all ELISA tests.

For this process, 96-well plates (Costar, Bethesda, MD, USA) were coated overnight with the purified hemagglutinins (25 ng/well) in 100 μL of coating buffer (0.05 mol/L carbonate/bicarbonate, pH 9.6) at 4°C and blocked with 300 μL of 1% bovine serum albumin for 2 h at 37°C. The amount of coating protein was optimized by a chessboard titration protocol (*20*). Serial-diluted serum samples (100 μL each) were added in duplicate for 1 h at 37°C. After 6 washes with 300 μL of phosphate-buffered saline containing 0.5% Tween-20, 100 μL of a 1:40,000 dilution of horseradish peroxidase–conjugated goat anti-human IgG (Sigma-Aldrich, St. Louis, MO, USA) was added to each well for 1 h at 37°C. The plates were then washed 6 times with 300 μL of phosphate-buffered saline containing 0.5% Tween-20 and incubated with 100 μL of substrate 3, 3′, 5, 5′-tetramethylbenzidine (Sigma-Aldrich) at 37°C for 15 min. The reactions were terminated by adding 50 μL of 2 mol/L hydrogen sulfate. The absorbance at 450 nm (A450) was determined for each serum sample. To evaluate the background signal, we used bovine serum albumin instead of human serum as the background signal control. The cutoff value was defined as twice the background signal (*21*). The antibody end-point titer was defined as the reciprocal of the highest dilution of serum that had a reading above the cutoff value.

### Antibody Avidity Analysis

For avidity analysis, an ELISA assay was performed as described (*22*). Serum was added at a dilution with an expected A450 of ≈1.0. Avidity was determined by incubating samples with urea at 4, 5, 6, and 7 mol/L for 30 min at room temperature before washing, followed by incubation with the horseradish peroxidase–conjugated goat anti-human IgG. The avidity index was defined as the ratio of the absorbance with urea to that without urea (*15**,**22*).

### Microneutralization Assay

Microneutralization assays were performed as described (*21*). In brief, serial dilutions of serum (starting at 1:10) were preincubated with 100 doses (50% tissue culture infective doses) of the influenza A/Anhui/1/2013 (H7N9) strain. After 2 h of incubation, the mixture was incubated with MDCK cells in 96-well plates (Costar). The virus/serum mixtures were removed after 2 h, and serum-free Dulbecco modified Eagle medium with 1 μg/mL of TPCK-trypsin (L-1-tosylamide-2-phenylethyl chloromethyl ketone-treated trypsin) (Sigma-Aldrich) was added to each well. The cytopathic effects were evaluated 96 h after incubation at 35°C in 5% CO_2_. For each antibody dilution, 4 duplicate wells were used. NAb titers were defined as the reciprocal of the highest serum dilution at which 50% of wells were protected.

### Hemagglutination Inhibition Assay

Hemagglutination inhibition (HI) assays were performed by using a β-propriolactone–inactivated A/Anhui/1/2013 (H7N9) strain according to World Health Organization protocol (www.who.int/influenza/gisrs_laboratory/cnic_serological_diagnosis_hai_a_h7n9.pdf). All HI assays were performed in V-bottom 96-well plates with 1% horse erythrocytes (*23*).

### Statistical Analyses

The Mann-Whitney U test and Student *t*-test were used for continuous variables. Serum IgG titers between groups were tested by using the Wilcoxon rank-sum test. HI titers were tested by using the Student *t*-test. All statistical calculations involving the geometric mean titers (GMTs) of antibodies were performed with log-transformed titers. Correlations between acute-phase serum antibodies against H1 and H3 and convalescent-phase serum antibodies against H7 were assessed by using the Spearman rank correlation coefficient test. p<0.05 was considered statistically significant.

## Results

A total of 21 patients with influenza A(H7N9) virus infection were enrolled in this study; 19 were from Shanghai and 2 were from Beijing, 15 were male and 6 were female, 19 were adults (47–89 years of age, median 68 years, mean 68.4 years), 1 was a 7-year-old girl, and 1 was a 4-year-old boy (Table 1). Only the 4-year-old boy experienced mild infection; 7 adults died. Patients were hospitalized 1–7 days after symptom onset. Major clinical manifestations included fever, cough, sputum, sore throat, myalgia, chills, dyspnea, and diarrhea. Most patients who died had complications of respiratory failure, multiple organ failure, or acute respiratory distress syndrome. Laboratory tests showed that some patients had liver or renal damage (Table 2). Major radiographic findings included pneumonia, increased markings, fuzzy patch lesions, and patch effusion shadows in lungs (Table 3). Of the 21 patients, 18 received antiviral treatment. The cycle threshold of real-time RT-PCR detection of subtype H7N9 virus ranged from 21.44 to 37.49 (median 30.48, mean 30.69) for the FluA (M gene), 24.34 to 38.00 (median 31.55, mean 31.92) for the H7, and 18.43 to 37.52 (median 29.65, mean 29.27) for the N9 tests, indicating a relatively low to medium viral load in most patients. Concomitant fungal infections occurred in 2 patients, and a concomitant *Acinetobacter baumannii* infection occurred in 1 patient (Table 2).

**Table 2:** Laboratory data for patients infected with influenza A(H7N9) virus, China, 2013*

Patient no.	H7N9 C_t_ for FluA, H7, N9 tests	Concomitant infection	ALT	AST	Creat	BUN	WBC	Temp	Serum collection, d†
1	28.47, 32.87, 32.55	No	39	77	74.3	NA	3.5	39.5	20
2	23.46, 24.34, 22.91	No	15	41	75.4	4.3	3.2	39.3	24
3	21.44, 34.76, 29.70	No	62	45	84.2	5.78	5.95	39.7	26
4	24.41, 25.91, 28.59	No	NA	NA	NA	NA	10.99	39.7	35
5	34.64, 31, Negative	No	36	40	65.0	6.8	4.7	39.0	35
6	31.99, 26.1, Negative	No	NA	NA	NA	NA	4.9	40.1	25, 31
7	29.89, 30.91, 27.63	*Candida albicans*	66	79	84.0	7.5	6.62	38.2	17, 27
8	30.26, 30.96, 30.6	No	31	35	84.0	41.0	4.1	38.9	25
9	37.49, 36.6, 36.95	No	33	78	102.0	43.0	5.64	39.5	24, 30
10	29.7, 26.43, 21.68	No	129	89	85.0	4.76	8.62	38.6	29
11	34.88, 36.18, 18.43	No	14	39	196.0	6.3	8.31	38.6	33
12	33.91, 33.66, Negative	No	86	144	83.0	3.6	7.11	39.0	37
13	30.48, 37.94, Negative	No	37	55	46.0	6.8	8.0	38.5	18
14	16.3, 16.1, 16.4	No	NA	NA	NA	NA	3.79	40.0	31
15	30.8, 33.0, 32.7	No	NA	NA	NA	NA	6.96	38.6	33
16	37.26, 38.48, 27.65	No	NA	NA	NA	NA	7.68	39.0	NA
17	30.73, 32.1, 28.85	No	100	30	NA	NA	11.2	39.5	NA
18	36.05, 35.75, 37.52	No	132	239	123.2	12.8	8.9	39.4	NA
19	27.21, 30.4, 30.62	*Acinetobacter baumannii*	25	19	88.7	2.85	7.93	39.8	NA
20	28.72, 27.76, 30.08	Fungus	NA	NA	NA	NA	9.5	38.5	NA
21	27.84, 35.23, 29.6	No	36	207	162.7	17.94	4.06	36.5	NA

*C_t_ , cycle threshold; ALT, alanine transaminase, U/L; AST, aspartate transaminase, U/L; Creat, creatinine, μmol/L; BUN, blood urea nitrogen, μmol/L; WBC, white blood cell (leuckocyte) count, ×10^9^/L; temp, body temperature, °C; NA, not available. †Convalescent-phase; days after symptom onset.

**Table 3:** Radiographic findings for patients infected with influenza A(H7N9) virus, China, 2013

Patient no.	Radiographic findings
1	Increased markings in both lungs, cloud floccule shadow in left lower zone
2	Increased markings in both lungs, visible small fuzzy patch shadow at right lower diaphragm
3	Pneumonia in left upper lung
4	Patch consolidation with dim edges in middle and lower zones of right lung
5	Pneumonia with partial consolidation in right lower lobe
6	Pneumonia in right lower lung
7	Increased markings in both lungs
8	Increased markings in both lungs. Visible patch lesions and strip lesions in 2 lower lobes
9	Patch lesions beside the right lung hilum, together with nodules and fuzzy strip shadows. Fuzzy patch lesions in left middle zone
10	Diffused effusion in both lungs
11	Increased marking in both lungs, fuzzy patch shadows in middle and upper lobes of right lung
12	No active lesion in either lung
13	Patch effusion shadows in right lower lung
14	Pneumonia in both lungs
15	Pneumonia in both lungs
16	Not applicable
17	Inflammation in the right lower lung
18	Pneumonia, increased markings in both lungs
19	Pneumonia, increased markings in both lungs
20	Fuzzy shadow in the left lower lung
21	Inflammation and consolidation in both lungs

**Figure 1:**
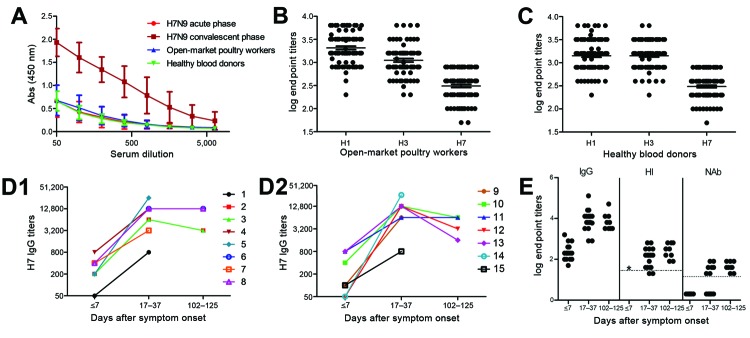
Serum antibodies (Abs) in patients infected with influenza A(H7N9) virus and in control populations (poultry-market workers and healthy blood donors), China, 2013. A) Dilution curves of IgG against subtype H7 in serum samples. Bars indicate SE. B and C) Titers of IgG against H7, H1, and H3 in poultry-market workers (B) and healthy blood donors (C). D) Increasing titers of IgG against subtype H7 after symptom onset in patients from whom paired serum samples were collected. E) Levels of IgG against H7, neutralizing antibodies (NAbs), and hemagglutination inhibition (HI) in serum samples after symptom onset. IgG against hemagglutinins of H7 and seasonal influenza A viruses (subtypes H1 and H3) in patients with subtype H7N9 virus infection and control populations were titrated by ELISA by using recombinant hemagglutinin antigens. NAbs were assessed by microneutralization assay the influenza A/Anhui/1/2013 (H7N9) strain. HI antibodies (Abs) were assessed by HI assay that used a β-propriolactone–inactivated influenza A/Anhui/1/2013 (H7N9) strain. B–E) Serum IgG, NAb, and HI titers were transformed to log_10_. For NAb-negative samples, titers of 2 were used for log_10_ transformation. Serum with titers >40 were considered HI positive for H7-specific antibody. The HI dotted line denotes a titer of log_10_40 = 1.60. Serum with titers >20 were considered NAb positive for H7-specific antibody. The NAb dotted line denotes a titer of log_10_20 = 1.30. *, not available.

A hemagglutinin ELISA was used to determine IgG titers (Figure 1). The end points for antibodies against hemagglutinin were obtained by determining A450 at a 2-fold serial dilution of each serum sample from patients and controls, starting at a dilution of 1:50 (Figure 1, panel A). High levels of IgG against H1 and H3 hemagglutinins were detected in the 2 control groups, as were GMT values of 2,070.40 and 1,118.10 in open-market poultry workers, and 1,476.80 and 1,448.50 in healthy blood donors, respectively. These findings suggest preexposure to seasonal influenza subtypes (H1 and H3) by both control groups (Figure 1, panels B, C). The low levels of IgG signal against H7 detected in the control groups (GMT 280.80 for poultry workers and 313.70 for healthy blood donors; median 400.00 for the 2 groups) (Figure 1, panels A–C) most likely resulted from antibody cross-reactivity, thus serving as the baseline of the assay for IgG against H7. The titers of IgG against H7 of the acute-phase serum samples (GMT 282.80, median 400.00) (Figure 1, panel A) did not differ significantly from those of the 2 control groups (p>0.05, Mann-Whitney U test).

The tested samples probably contained no preexisting antibodies against the H7 subtype. In contrast, IgG titers in the convalescent-phase samples increased greatly on days 17–37 (GMT 7,412.40, median 9,600.00) (Figure 1, panels A, D, E); GMT was 26.2-fold higher for convalescent-phase than for acute-phase samples. To monitor the antibody dynamics in patients, we further analyzed IgG titers against H7 after patients recovered from subtype H7N9 virus infection. No obvious changes were detected in serum samples collected 102–125 days after symptom onset (GMT 7,465.80, median 6,400.00) (p>0.05, Wilcoxon rank-sum test) (Figure 1, panel D).

NAbs against subtype H7N9 virus were not detectable in serum from poultry-market workers or healthy blood donors (data not shown). No NAbs were detected in samples collected before day 28, although they were detected in most samples (7 of 9 patients) collected 29–37 days after symptom onset (NAb titers ranged from 20 to 80, GMT 40, median 40) (Figure 1, panel E). This finding differs from that observed for influenza A(H1N1)pdm09 and subtype H5N1 virus infections, in which NAbs were detected 14–21 days after symptom onset (*16**,**24*). NAbs were positive in all serum samples collected from the 9 recovered patients 102–125 days after symptom onset (NAb titers 20–80, GMT 40, median 40). However, the NAb titers did not significantly increase compared with those of the convalescent-phase samples collected at 17–37 days (Figure 1, panel E) (p = 0.906, Student *t*-test).

Because we lacked sufficient blood samples, we did not analyze HI in acute-phase serum. HI titers in convalescent-phase serum were measured in parallel to those in serum obtained at 102–125 days. HI titers ranged from 20 to 640 (GMT 117.60, median 160.00) for the convalescent-phase serum collected at 17–37 days and from 80 to 640 (GMT 260.00, median = 320.00) for serum collected at 102–125 days. The differences were not significant (p = 0.886, Student *t-*test) (Figure 1, panel E).

**Figure 2:**
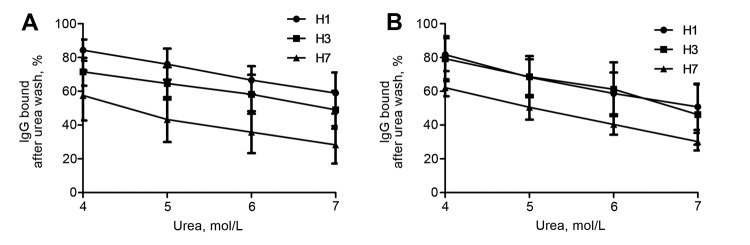
Avidity analysis of antibodies in patients infected with influenza A(H7N9) virus, China, 2013. The avidities of IgG against influenza viruses were determined by ELISA assay with 4–7 mol/L urea. Shown are avidities of IgG against H1, H3, and H7 hemagglutinin of convalescent-phase serum samples collected 17–37 days (A) and 102–125 days (B) after symptom onset. Bars indicate SE.

Further characterization of the antibody response by avidity analysis showed that the percentage of IgG bound to H7 was much lower than that bound to H1 or H3 in convalescent-phase serum collected 17–37 days after symptom onset when treated with different urea concentrations in the ELISA assay (p<0.05, Student *t-*test) (Figure 2, panel A). Similar results were observed for serum collected 102–125 days after symptom onset (Figure 2, panel B; p>0.05, Student *t-*test). These data suggest that IgG avidity to H7 is significantly lower than that to seasonal influenza A viruses.

**Figure 3:**
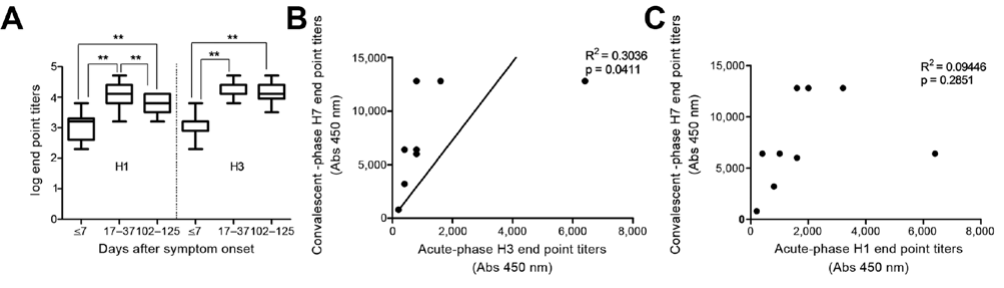
Association between antibody responses against H7 and seasonal subtypes in patients infected with influenza A(H7N9) virus, China. A) Levels of IgG against H1 and H3 in serum samples after symptom onset. IgG in samples taken at acute-phase (≤7 days), convalescent-phase (17–37days,) and 102–125 days after symptom onset were titrated by ELISA with recombinant H1 and H3 hemagglutinin antigens, respectively. IgG titers were transformed to log_10_. Bars indicate SE. B and C) Correlation between IgG against H3 (B) and H1 (C) in acute-phase serum and against H7 in convalescent-phase serum. **p<0.01.

In parallel to IgG detection for control groups, we also evaluated IgG levels against seasonal influenza virus hemagglutinins H1 and H3 by ELISA of the 15 paired serum samples (Figure 3, panel A). IgG against H1 and H3 was detected in the acute-phase serum (GMT 1,114.00 and 933.30, median 1,600.00 and 800.00, respectively), indicating preexposure of these patients to seasonal influenza virus. IgG titers did not differ significantly between acute-phase serum from patients with subtype H7N9 infection (most were older persons) and that from control groups (p>0.05, Mann-Whitney U test; Figure 1, panels B, C). This finding differs from that observed for patients with A(H1N1)pdm09virus infections, among whom high levels of HI antibody against A(H1N1)pdm09virus in older populations has been reported (*25**,**26*). This disparity might result from different detection methods (IgG vs. HI) and different times and doses of exposure to influenza virus.

Of note, a boost of IgG titers against H1 and H3 in convalescent-phase serum collected at 17–37 days (GMT 13,128.50 and 16,345.40, median 12,800.00 and 16,400.00, respectively) was observed, compared with that of the acute-phase serum, in which a boost was not detected (p<0.01, Wilcoxon rank-sum test). IgG titers against H1 were lower in serum collected at 102–125 days (GMT 5,486.40, median 6,400.00) than in serum collected at 17–37 days (p<0.01, Wilcoxon rank-sum test) (Figure 3, panel A) but were still higher than those in the acute-phase serum (p<0.01, Wilcoxon rank-sum test). Titers of IgG against H3 did not obviously change in serum collected at 102–125 days (GMT 10,972.70, median 12,800.00) compared with that collected at 17–37 days (p>0.05, Wilcoxon rank-sum test) (Figure 3, panel A). Moreover, a significant correlation (R^2^ = 3036, p = 0.0411) was observed between the levels of IgG against H3 in acute-phase serum and that against H7 in convalescent-phase serum (Figure 3, panel B) but not between the levels of IgG against H1 in acute-phase serum and IgG against H7 in convalescent-phase serum (R^2^ = 0.009446, p = 0.2851) (Figure 3, panel C), indicating that there is a heterologous boost of IgG against H3 by H7 hemagglutinin.

**Figure 4:**
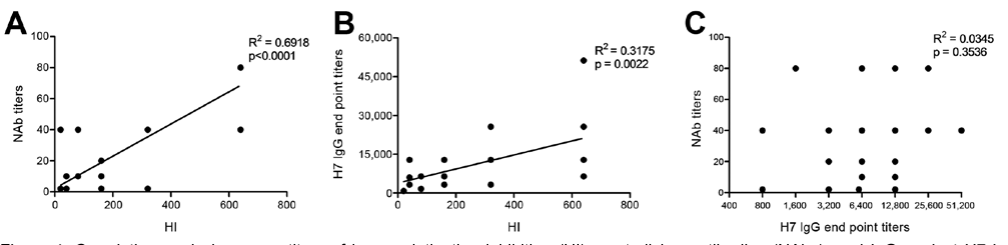
Correlation analysis among titers of hemagglutination inhibition (HI), neutralizing antibodies (NAbs), and IgG against H7 in patients infected with influenza A(H7N9) virus, China, 2013. A) NAb vs. HI. B) IgG against H7 vs. HI. C) NAb vs. IgG against H7.

Further evaluation that used data obtained from the serum collected 17–37 days and 102–125 days after symptom onset revealed a significant correlation between titers of HI and NAb (R^2^ = 0.6918, p<0.0001) and between titers of HI and IgG against H7 (R^2^ = 0.3175, p = 0.0022) (Figure 4, panels A, B). However, no correlation was found between titers of IgG against H7 and NAb (R^2^ = 0.0345, p = 0.3536) (Figure 4, panel C), indicating that titers of IgG against H7 might may not be an ideal indicator for protective immunity against infection with influenza A(H7N9) virus.

## Discussion

The protective antibody response against influenza A(H7N9) virus was relatively weak; the NAb response was lower than that for influenza A(H1N1)pdm09 and H5N1 viruses. Studies have shown that the GMT of convalescent-phase NAbs against A(H1N1)pdm09virus is 1:101.1 at 21–42 days after symptom onset (*24*), but the NAb titers against subtype H5N1 virus range from 80 to 2,560 at 14 days after symptom onset in convalescent-phase serum samples (*16*). Moreover, the serum IgG avidity for H7 was lower than that for H1 and H3. Of note, a significant boost of titers of IgG against H3 in convalescent-phase serum and a correlation between the level of IgG against H3 in the acute-phase serum and the IgG level against H7 in the convalescent-phase serum were observed.

Our findings raise several questions about the role of humoral responses to subtype H7N9 virus infection and disease outcome. Does the relatively weak NAb response of the host against subtype H7N9 play a role in the severity and duration of infections? And does the low avidity of IgG against H7 hemagglutinin correlate with subtype H7N9 pathogenesis? 

With regard to the first question, it has been reported that for other influenza A viruses, the presence of NAbs correlates with recovery time and the outcomes of the disease (*24*). We speculate that the relatively weak NAb response against subtype H7N9 might directly contribute to the severity of the symptoms. In this regard, we note that 1 patient, for whom no Nabs were detectable in convalescent-phase serum at days 17 and 27, died and that another patient, for whom NAb titer was low (1:20) on day 31, also died, although titers of IgG against H7, H1, and H3 in both patients were very high.

There are several possible explanations for the relatively low NAb response. Immunogenicity of subtype H7N9 virus is probably weaker than that of influenza subtype H5N1 and A(H1N1)pdm09 viruses. An immune-informatic analysis predicted that the T-cell epitope contents are low in subtype H7N9 proteins, probably leading to a lower immune response against subtype H7N9 virus (*27*). Furthermore, it has been reported that the humoral response to subtype H7N7 vaccine is lower than that to subtypes H5N1 and H9N2 after vaccination with a dose that should have stimulated effective immune response (*28*). Another reason for the lower NAb response could be inefficient T-cell helper response. The isotype switch of antibody production, such as that from IgM to IgG, as well as the process of antibody affinity maturation, requires T-cell help (*29*). This point should be investigated in future studies, such as analysis of peripheral blood mononuclear cells, which were not available in our study. Patients’ immune status might also provide an explanation. Most patients in this study had underlying diseases, which might attenuate the immune responses (*30*). In addition, age can also play a role in attenuated immunity; the severity of infection with subtype H7N9 virus increases with age (*2*). It is possible that the relatively low NAb response against subtype H7N9 infection is caused by several of the aforementioned factors.

With regard to the second question, whether the low avidity of IgG against H7 hemagglutinin correlates with subtype H7N9 pathogenesis, previous studies demonstrated that low-affinity antibodies against A(H1N1)pdm09 virus could form pathogenic immune complexes to impair multiple organ functions and were associated with disease outcome (*21*). In this study, we found that although high titers of IgG against H7 developed in patients infected with subtype H7N9 virus, binding avidity to this subtype is much lower than that to seasonal influenza A viruses. In particular, we found that among tested patients, although IgG against H7 was highest (1:25,600) among the 2 deceased patients from whom paired serum samples were obtained, IgG avidity for subtype H7N9 was also very low. Whether the nonprotective, low-avidity antibody response plays a major role in the pathogenesis of subtype H7N9 virus in addition to the relatively weak NAb response needs to be elucidated.

In our study, we observed a significant boost of antibody against H3 and H1 in patient serum samples. Titers of IgG against H3 in acute-phase serum were correlated with those against H7 in convalescent-phase serum. Perhaps subtype H7N9 virus infection triggered a cross-reactive response between H7 and H3 or H1 hemagglutinins. Primary infection with influenza virus can lead to a heterosubtypic hemagglutinin antibody response (*31*). Moreover, hemagglutinin stalks are structurally conserved within each hemagglutinin subgroup. The induction of anti-stalk antibodies during influenza virus infection provides the cross-reactivity and protection against infections by different influenza A virus subtypes (*17**, **32**–**39*). However, in our study, the cross-reactivity of H7 versus H1 was lower than that of H7 versus H3 (Figure 3). Because H3 and H7 subtypes belong to subgroup 2 of hemagglutinin, and H1 hemagglutinin belongs to subgroup 1, it is reasonable that such cross-reactivity is stronger between H7 and H3 than between H7 and H1 and H5 (*40*). Because a substantial proportion of persons all over the world have experienced H3N2 virus infection, the effects of such boost responses in infection with subtype H7N9 should be further investigated (*17*).

Our main study limitation was the small number of patients for whom paired serum samples were available. These few paired samples were insufficient for certain analyses, such as comparing differences across age groups and sex. Another limitation was the timing of convalescent-phase serum sample collection, which occurred immediately before hospital discharge. The varied sampling times might have influenced the accurate identification of NAb occurrence and NAb titer comparisons among patients. In addition, clinical information was incomplete for some patients; and given the small sample size, we were unable to correlate antibody responses with disease severity.

In summary, our findings indicate a relatively weak protective antibody response against influenza A(H7N9) virus in tested patients. This low response might provide insights useful for potential vaccine development against subtype H7N9; multiple vaccinations might be needed to achieve protective immunity.
